# Effects of extremely low frequency electromagnetic fields on the tumor cell inhibition and the possible mechanism

**DOI:** 10.1038/s41598-023-34144-5

**Published:** 2023-04-28

**Authors:** Jie Sun, Yingying Tong, Yu Jia, Xu Jia, Hua Wang, Yang Chen, Jiamin Wu, Weiyang Jin, Zheng Ma, Kai Cao, Xiangdong Li, Zhonglin Chen, Guanghua Yang

**Affiliations:** 1 International Research Center for Biological Sciences, Ministry of Science and Technology, Shanghai Ocean University, Shanghai, 201306 China; 2 National Aquatic Animal Pathogen Collection Center, Shanghai Ocean University, Shanghai, 201306 China; 3 Aquatic Animal Genetics and Breeding Center, Shanghai Ocean University, Shanghai, 201306 China; 4 Shanghai Telebio Biomedical Co., Ltd, Shanghai, China; 5 Zhejiang Huayi Health Industry Development Co., Ltd, Hangzhou, China; 6 Huisi Anpu Medical System Co., Ltd, Qinhuangdao, China

**Keywords:** Biophysics, Cell biology, Physics

## Abstract

Low-frequency magnetic fields exert a significant inhibitory effect on tumor growth and have been developed as a therapeutic modality. However, the effect of a low-frequency magnetic field on the interaction between cells is still poorly understood. This study aimed to preliminarily evaluate the direct effect of magnetic field ditectely on cultured cells and indirect effect mediated by cell-environment (conditioned medium). 293 T cells, Hepg2 cells, A549 cells have been cultured at 37 ± 0.18 °C in presence of an extremely low-frequency magnetic field of 20 Hz, 5-mT. The adherent tumor cells were more sensitive to magnetic field inhibition in the original environment (conditioned medium) with adherence inhibition rate for Hepg2 and A549 estimated at 18% and 30% respectively. The inhibition effect was suppressed when the suspended cells separated or clump density at a low density. The nontumor cell lines showed no inhibitory effect on exposure to a low-frequency magnetic field. The intracellular ion fluorescence (IIF) showed that the magnetic field significantly altered the membrane potential, indicating hyperpolarization of the adherent cells (ΔIIF 293 T cells: − 25%, ΔIIF Hepg2 cells: − 20% and ΔIIF A549 cells: − 13%) and depolarization of the suspended cells (ΔIIF Raji cells: + 9%). In addition, the conditioned media collected after magnetic field exposure acted on unexposed tumor cells and caused inhibition. Our findings might provide a basis for the mechanism of magnetic field interaction between cells and cell environment in the future.

## Introduction

Low-frequency magnetic fields exert noninvasive, nonionizing, and nonthermal effects on cells and tissues. They enhance the cellular oxidative stress response and regulate the apoptotic signaling pathway, changing the intracellular Ca^2+^ concentration to induce apoptosis^1–3^. They are widely used to treat tumors and neuropsychiatric and bone diseases. In vivo studies in this field have shown that low-frequency magnetic fields inhibit the proliferation of tumor cells and prolong their survival^4–11^.

In most reports on using magnetic fields as a combination therapy, extremely low-frequency magnetic fields enhance the efficacy of antitumor drugs^12–15^. A combination of an extremely low-frequency magnetic field with paclitaxel in treating mouse cancer revealed that the magnetic field increased the execution lethality of paclitaxel^16^. The cell membrane permeability was altered, and the therapeutic effect of cisplatin was significantly enhanced at an extremely low-frequency magnetic field of 10 mT combined with cisplatin^17^. However, Gellrich found that the low-frequency magnetic field could not enhance the therapeutic effect of cetuximab, which might be related to the conformational change in the molecular surface receptor^18^.

In most in vitro experiments, the low-frequency magnetic field showed a significant inhibitory effect on tumor cells^2,3,19–23^ and did not affect the growth of normal cells^2,24^. A report found that the magnetic field affected the surface of the tumor membrane, thus influencing tumor proliferation^25^. However, some reports showed that the proliferation of tumor cells slightly increased under the low-frequency magnetic field^26^.

At present, it is believed that magnetic fields can significantly inhibit tumor growth, and the inhibitory effect has a positive correlation with time and intensity. Meanwhile, the production of reactive oxygen species (ROS) is an inevitable phenomenon considered to be the key to the inhibitory effect of the magnetic field^3^. However, the exact mechanism is unclear. In the development of antineoplastic therapies, the inhibitory effect of the magnetic field on tumor growth is a significant attribute to the clinical performance of many existing technologies.

Many experiments have been conducted on the differences in magnetic field settings, but little research has been done on the effect of the difference in the magnetic field on the tumor environment and the possible inhibition mechanism, except ROS. In this study based on the effect of a magnetic field on the intercellular environment and intercellular structure (the form of natural contact between cells and the form of human interference), the cells were cultured in vitro. The study found that the state of intercellular aggregation was a necessary phenomenon for magnetic inhibition. At the same time, during cell proliferation, one or several related substrates were released in the conditioned medium, which might act together with the magnetic field to achieve the effect of magnetic field inhibition.

In this experiment, a magnetic field of 5 mT and 20 Hz was used as the sole background. In previous experiments, the magnetic field intensity was not fixed, and it often did not directly contact cells or could not be placed in an incubator. The simple magnetic field generator designed in this study could be in direct contact with cells and placed in an incubator under stable conditions of temperature and CO_2_. Our magnetic field generator also had disadvantages, that is, when a magnetic field was generated, it also generated heat. Based on the intensity design of Crocetti^19^, a magnetic field generator was designed to stabilize the heat through heat dissipation.

## Results

### Magnetic field inhibited the adherent tumor cells, which was influenced by the difference in the culture environment (conditioned medium)

**Figure 1:**
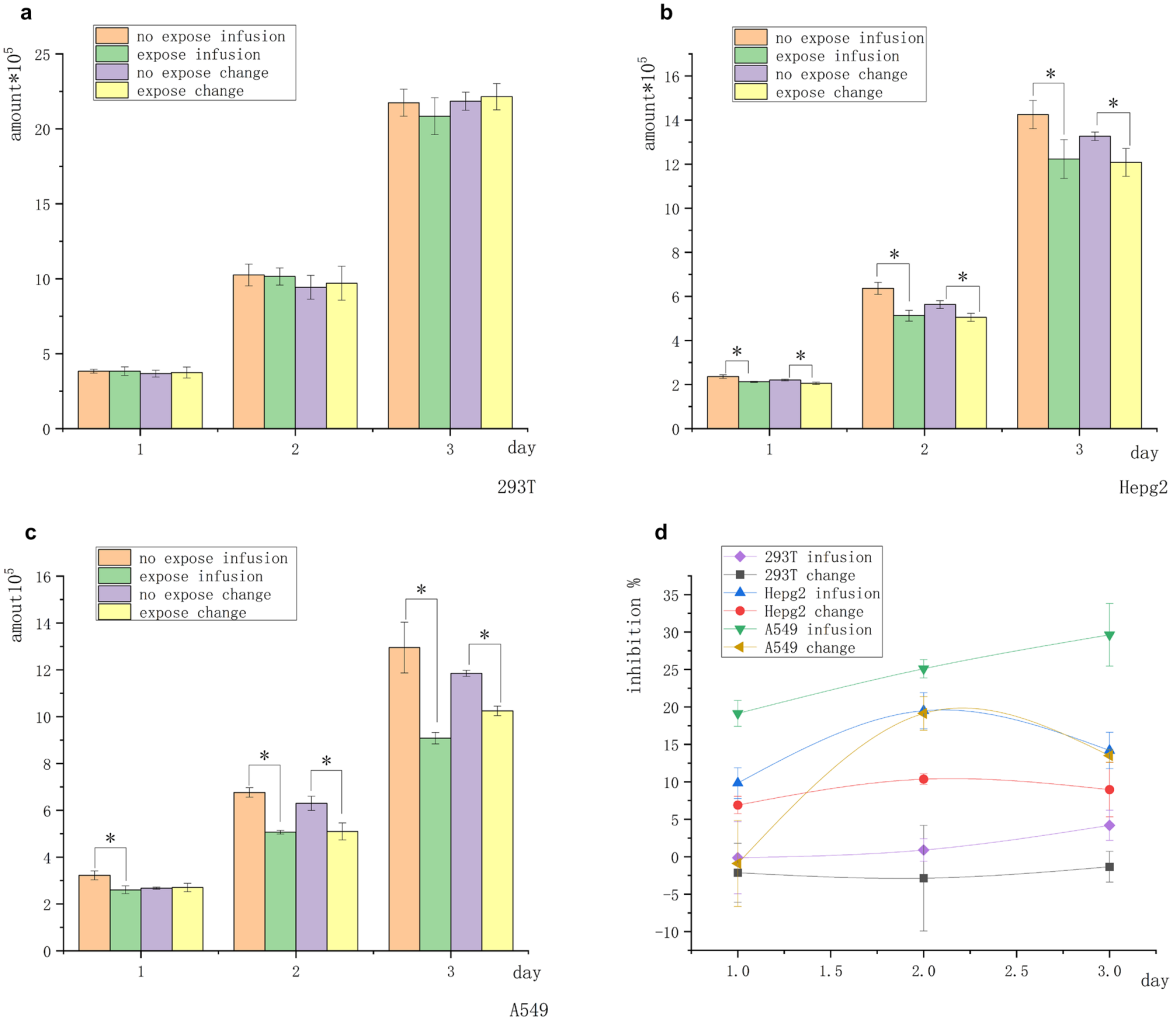
Difference in the environment (conditioned medium) before exposure affected the inhibitory effect of the magnetic field on adherent cells. (**a**) No significant difference was found in the number of 293 T cells in 3 days. (**b**) Number of Hepg2 cells in the unexposed and exposed groups was significantly different. (**c**) Number of A549 cells in the unexposed and exposed groups was significantly different. (**d**) Cell inhibition curve. The cell inhibition rate in the infusion group was more obvious than that in the change group, and the normal cell inhibition rate was not of statistical significance (^*^P < 0.05, versus the no-exposure control group).

The treatment groups were divided into two groups before exposure. Infusion group: Prior to daily exposure, 500 µL of the fresh medium was slowly added with a pipettor to the pore wall. The conditioned culture medium was a mixture of various substances (medium incubated overnight after passage and lamination) and a fresh medium for exponential growth. Change group: Prior to daily exposure, a pipette was applied to the pore wall to remove most of the medium (almost all), which was replaced with a medium of the same volume as the “infusion” group. The conditioned medium was completely fresh with no or minimal secretions. The difference between the two groups was the composition of the conditioned medium: the composition in the “infusion group” was more complex, while the composition in the “change group” was closer to that of the unused medium. Normal human renal epithelial cells 293 T, human liver cancer cells Hepg2, and human nonsmall-cell lung cancer cells A549 were processed independently through culture medium “infusion” or “change” and exposed to the 5-mT intensity of the magnetic field for 2 h each day for a total of 3 days. The initial number of all cells was 2 × 10^5^. Figure 1a shows that the nontumor cell line 293 T was not inhibited by the magnetic field in the “infusion group” and the “change group”. Figures 1b and c shows that the number of Hepg2 and A549 cells exposed to the magnetic field was significantly lower than that of cells in the unexposed control group. Both tumor cell lines Hepg2 and A549 were inhibited by the magnetic field (the highest inhibition rate of Hepg2 was about 18%, and that of A549 was about 30%). The tumor cells in the “infusion group” showed inhibition on day 1, while the cells in the “change group” showed no significant inhibition. The inhibition trend in the “infusion group” was significantly stronger than that in the “change group”. The inhibitory effect in the “infusion group” (A549) positively correlated with exposure duration (Fig. 1d). These results indicated that the tumor cells were more sensitive to the magnetic field in the conditioned medium (microenvironment) modified by autocrine and paracrine signals.

### Spontaneous aggregation of suspended tumor cells was destroyed, and magnetic inhibition disappeared

We investigated whether a nutrient loss in the conditioned medium, excessive cell density, and interference with magnetic field inhibition by trypsin could affect the longer duration of magnetic inhibition. In particular, we performed the experiment using suspended lymphoma Raji cells. In natural culture, the suspended cells spontaneously gathered into clusters. The structure of such clusters was inevitably destroyed when the centrifuged cells were replaced with a conditioned medium in the change group. We also destroyed the cluster structure in the infusion group to ensure the consistency of experimental conditions. The suspended cells were infused or changed with the culture medium and exposed to a 5-mT magnetic field for 2 h daily for 6 days. Infusion group: Prior to daily exposure, 500 µL of the fresh medium was slowly added with a pipettor to the pore wall, and the cells were blown with a pipette gun to separate them in suspension and destroy the agglomerative structure. Change group: Prior to daily exposure, the cell suspension was aspirated and centrifuged at 1200 rpm to remove the supernatant, which was then replaced with a medium of the same volume as the “infusion” group. On day 3, the conditioned medium was completely replaced in the infusion group, while transferring the cells from both groups to larger containers. The suspended tumor cells were cultured in vitro without trypsin and easily transferred to larger containers, avoiding trypsin damage. Under such conditions, neither group showed significant inhibition compared with the no-exposure control group.

**Figure 2:**
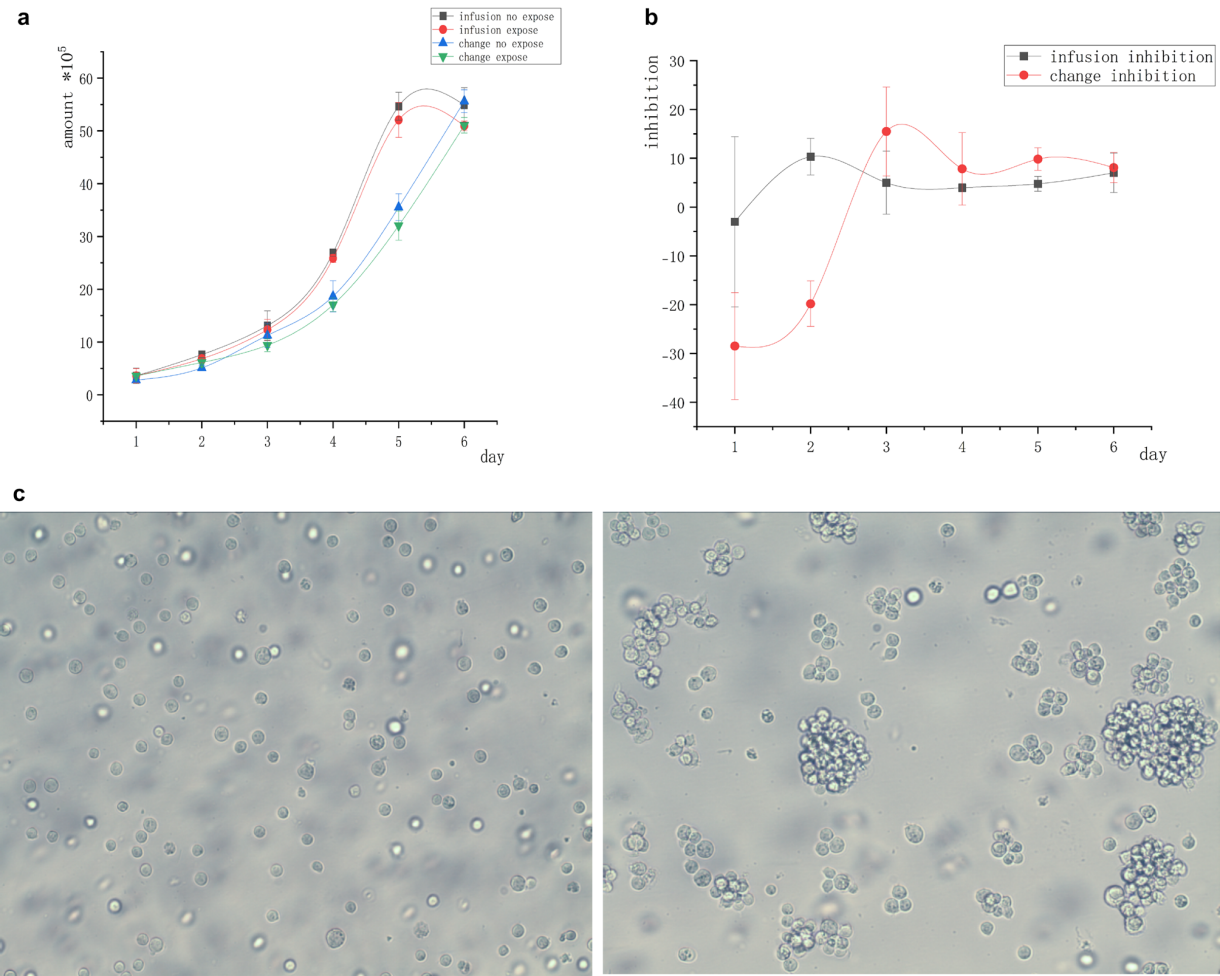
Cell growth curve of freely suspended Raji tumor cells in the infusion and change groups. (**a**) Cell inhibition curves under different degrees of the freshness of the culture medium. Under the condition of cell dispersion, no significant difference was found between the number of cells exposed to the magnetic field and those in the unexposed control group. (**b)** Inhibition rate curves under the condition of cell dispersion; the tumor cells were not inhibited on magnetic field exposure under different degrees of the freshness of the culture medium. (**c**) Spontaneous cell clustering (right) and artificial separation (left).

The initial number of all cells was 2 × 10^5^. Figure 2a shows no significant difference in the number of cells in the “infusion group” and “change group” after 6 days of magnetic field exposure compared with the no-exposure control group. Figure 2b shows that the magnetic field inhibition was not obvious after the aggregation structure disappeared.

As shown in Fig. 2a, the number of cells in the “infusion group” was significantly higher than that in the “change group” on day 4 (after replacing the large container). The “infusion group” environment was more suitable for the growth of suspended tumor cells.

Figure 2c shows suspended cells in separate and natural suspended states. It was seen that most cells were single-celled instead of multi-celled clusters after the destruction of the cluster structure.

### Spontaneous aggregation of suspended tumor cells was retained, and magnetic inhibition appeared

In the earlier experiment on Raji lymphoma cells, the spontaneous aggregation of suspended tumor cells was destroyed and magnetic inhibition disappeared. We demonstrated that the magnetic field did not exert any inhibitory effect on freely suspended cancer cells. In this group of experiments, we examined whether cell contact played a role in the effect of magnetic inhibition on tumor growth. Aggregated Raji cells in clusters were studied in a 9-day continuous-exposure experiment. The cells were transferred to a larger container, while the culture medium was changed every 3 days. “Agglomerate” group: No procedures were performed except for medium replacement every 3 days and transfer to larger containers. “Dispersed” group: Prior to daily exposure, the cells were blown with a pipette gun to separate the cells in suspension and destroy the agglomerative structure.

**Figure 3:**
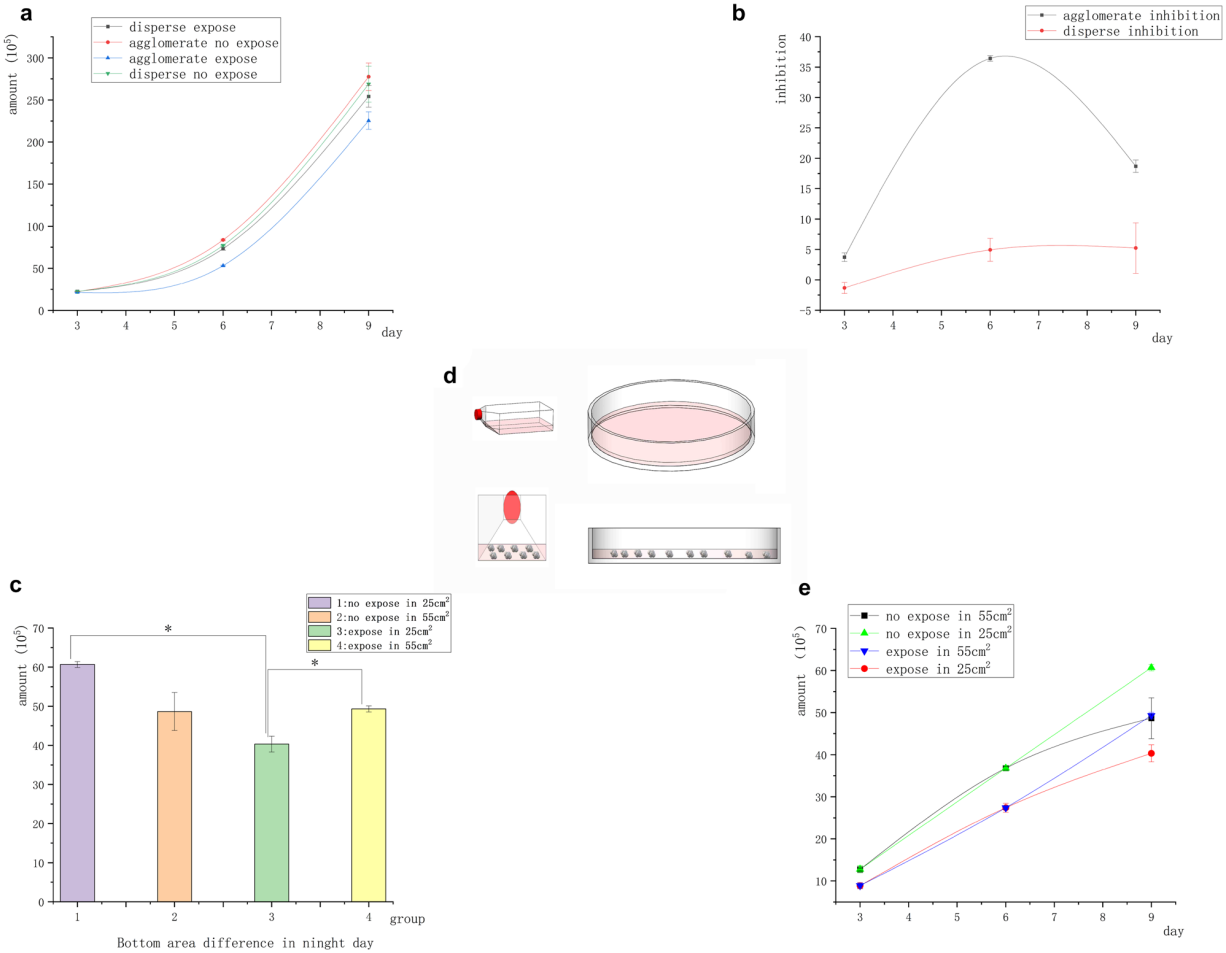
Agglomerate and dispersed inhibition of suspended Raji tumor cells. (**a**) Growth curve of suspended cells with different contact structures showed that the number of cells in the group exposed to the magnetic field was significantly different from that in the group not exposed to the magnetic field under the condition of cell aggregation. However, no significant difference was found between the group exposed to the magnetic field and the group not exposed to the magnetic field under the condition of cell dispersion. (**b**) Structure difference inhibition curve: The clustered cells had significant inhibition, but the dispersed cells had no inhibition. (**c**) On day 9, under the same volume and different basal areas, the number of cells in the group with smaller basal areas significantly reduced (^*^P < 0.05, vs the no-exposure control group; ^*^P < 0.05, vs the group with different bottle area) (**d**) As shown in the figure, the cells clustered more closely in the culture flask, while the cells in the Petri dish were scattered at the bottom due to the low liquid level. (**e)** Differential growth curve of the bottom area of the culture vessel on day 9.

The initial number of all cells was 2 × 10^5^. Figure 3a shows that the number of Raji cells under suspension aggregation in the magnetic field was significantly lower than that in the unexposed control group, whereas the number of Raji cells under suspension separation in the magnetic field showed no significant differences compared with the unexposed control. Figure 3b shows the difference in the magnetic field inhibition rate between the two groups of cells (suspension aggregation and suspension separation). It indicated that Raji cells were inhibited by the magnetic field under the condition of suspension aggregation. Meanwhile, Raji cells were not inhibited by the magnetic field under the suspension separation condition. Raji cells were less sensitive to magnetic inhibition than adherent cells, and the inhibition rate of Raji cells on day 6 was similar to that of A549 cells on day 3. In the absence of the magnetic field, the number of Raji cells in the suspended aggregation group was significantly higher than that in the suspended separation group. In contrast, on exposure to the magnetic field, the number of Raji cells in the suspended separation group was significantly higher than that in the suspended aggregation group. However, on day 9, the magnetic inhibition decreased or disappeared. We repeated the experiment to determine the reasons for the disappearance of the inhibition rate on day 9. We took the area at the bottom of the container as the control to investigate whether the differences in cell cluster density caused the reduction of magnetic inhibition (less contact between cells), and exposed the cells in the clustered state to a 5-mT, 20 Hz magnetic field for 9 days. The conditioned medium was changed every 3 days, and the large container was replaced. The operation remained unchanged for the first 6 days. On the sixth day of transfer, the cells were divided into containers with different base sizes [10-cm Petri (55-cm^2^) dish and 25-cm^2^ culture flask]. The only change was that the cells were transferred on day 6, using the 10-cm Petri dish (55 cm^2^) and 25-cm^2^ flask (under different basal areas, the cell masses were more concentrated) as controls. Surprisingly, the inhibition rate of Raji cells in the 25-cm^2^ flask was as high as 36% on day 9. The inhibition of Raji cells in the 10-cm Petri dish (55 cm^2^) disappeared on day 9 (Fig. 3c). The results were consistent with previous findings. The reason for this result should be related to the closeness of the cells (Fig. 3d).

### Membrane potential was related to magnetic field inhibition

**Figure 4:**
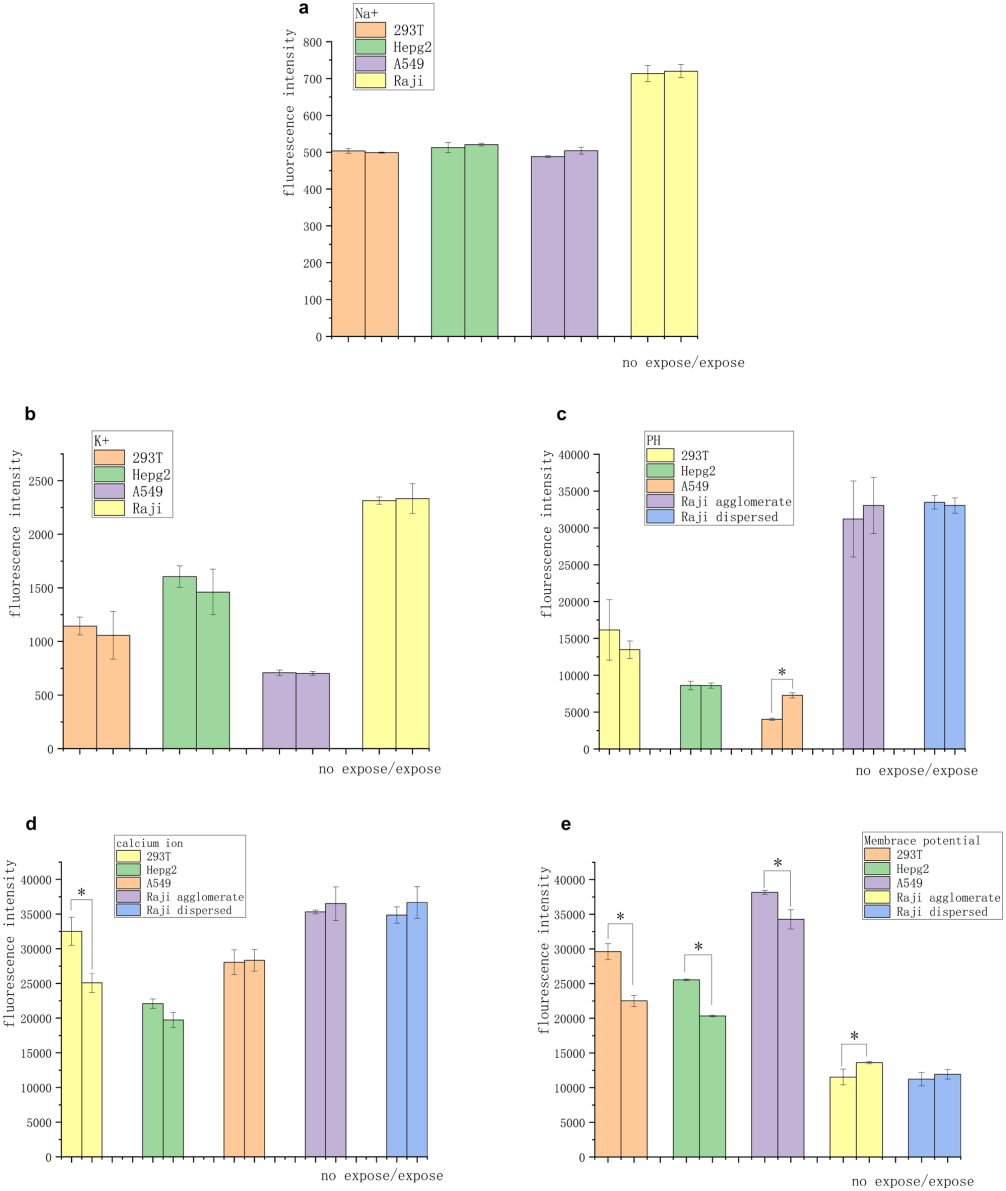
Differences in ionic strength and membrane potential of cells under 3-day 5-mT 20-Hz magnetic field exposure. (**a**) No significant difference was found in the fluorescence intensity of sodium ions after 3 days of exposure. (**b**) No significant difference was found in the fluorescence intensity of potassium ions after sodium ion exposure for 3 days. (**c)** After 3 days of exposure, no significant difference was found in the fluorescence intensity in all groups of cells except A549. (**d**) Calcium ion concentration in the 293 T cells decreased, while no significant difference in the calcium ion concentration was observed in the other groups. (**e**) Adherent cells showed significant hyperpolarization, tumor cell agglomerates showed significant depolarization, and the freely suspended tumor cells showed no significant depolarization. These changes corresponded to the changes in intracellular calcium ion concentrations (^*^P < 0.05, vs the no-exposure control group).

The experiments on suspended tumor cells showed that the inhibitory effect of magnetic field on cancer cells was accomplished through contact and communication between cells, having implications for signal transmission between cells and ionic changes in the cell microenvironment. Calcium, sodium, potassium, and pH kits Calbryte 520 AM, SBFI AM, PBFI AM, and BCECF AM were used to observe the changes in intracellular free ions of four kinds of cells after 3 days of magnetic exposure so as to investigate whether the magnetic field suppression was associated with ionic signaling. No changes in intracellular sodium and potassium ion concentrations were observed in normal or tumor cells (Fig. 4a and b). Also, no significant difference was found in pH fluorescence intensity in all groups of cells except A549 (Fig. 4c). Normal 293 T cells showed a significant decrease in the intracellular free calcium ion concentration. The solid tumor cells showed no significant change, while the suspended tumor cells showed a slight increase in the calcium ion concentration (Fig. 4d).

A change in the calcium ionic concentration is usually reflected by a change in membrane potential. The membrane potential kit DiBAC4 (3) was used to observe the exposed cells on day 3 with ΔIIF corresponding to ration of the intracellular ion fluorescence for Day 3 and initially. The adherent cells showed significant hyperpolarization (ΔIIF 293 T cells: − 25%, ΔIIF Hepg2 cells: − 20% and ΔIIF A549 cells: − 13%). The tumor cell agglomerates showed significant depolarization (ΔIIF Raji cells: + 9%). The free suspended tumor cells showed no significant depolarization (Fig. 4e). Figures S1–S3 show flow cytometry data.

### Cells secreted substances in the conditioned medium, which interacted with the magnetic field to inhibit tumor cells; the substance had universal expression and was tumor specific

**Figure 5:**
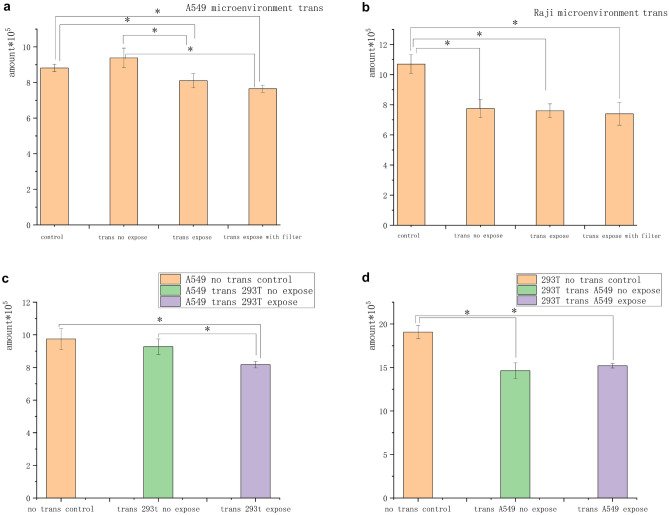
Conditioned medium inhibited tumor cell growth. (**a**) After A549 cells were exposed to the magnetic field, the conditioned medium was filtered and replaced with the unexposed conditioned medium. The exposed conditioned medium A549 had an inhibitory effect on the unexposed A549 cells. (**b**) After Raji cells were exposed to the magnetic field, the conditioned medium was filtered and replaced with the unexposed conditioned medium. No difference was found in the number of cells between the change groups, but the number of cells in the change group was significantly lower than that in the untreated control group. (**c**) Number of A549 cells transferred from the exposed 293 T cell conditioned medium significantly reduced compared with that from the untreated control and unexposed conditioned medium. (**d**) No significant difference was found in the number of 293 T cells in the conditioned medium of A549 cells after exposure compared with that in the untreated and unexposed groups (^*^P < 0.05, vs the no-exposure control group; ^*^P < 0.05,vs the no-transfer control group).

A549 and Raji cells in agglomerates were exposed independently to the 5-mT magnetic field for 3 h to determine whether the magnetic field inhibition was related to a change in the conditioned medium in the state of cell aggregation. The conditioned medium was then transferred to feed unexposed cells of the same species for 2 days. The number of cells in the A549 “transfer” group was 4 × 10^5^. The number of cells in the Raji “transfer” group was 8 × 10^5^. The number of cells in the A549 “be transferred” group was 2 × 10^5^. The number of cells in the Raji “be transferred” group was 4 × 10^5^. Under these conditions, the cells in the A549 “be transferred” group were significantly inhibited at a rate of approximately 10%, which was nearly half of that of the cells with direct exposure on day 1 in the fluid infusion group. The cells in the Raji “be transferred” group showed no inhibition, but the cells in the transfer groups were inhibited compared with those in the unexposed control group (Fig. 5a and b).

In addition, A549 and 293 T cells were exposed to the 5-mT magnetic field for 3 h to determine whether this conditioned medium was unique to tumor cells and inhibited normal cells. The exposed culture media were then transferred to feed unexposed cells of the different species for 2 days. The results showed that A549 cells were significantly inhibited in 293 T culture media. The cells in the 293 T transfer group showed no inhibition, but the cells in all transfer groups were inhibited compared with those in the unexposed control group (Fig. 5c and d).

## Discussion

Magnetic field exposure has the potential to serve as an advanced strategy in cancer management. ROS produced by cells using this method appear to be the key to tumor growth inhibition^3^. We proved that the main mechanism was not only related to ROS but also closely related to the contact between cells. We speculated that the magnetic field exposure changed the secretion of certain substrates, leading to the changes in signaling and thus restoring tumor cell contact inhibition. Based on the experimental results and previous experiments, four properties of magnetic field suppression were summarized.

1. The experiments on the magnetic field inhibition of cells, especially Raji cells, showed that the close contact between cells directly determined whether the inhibition effect existed. As shown in Figs. 2a, 3a, and c, suspended aggregation accelerated the growth of suspended tumor cells, while the magnetic field exposure inhibited the growth of tumor cells. As shown in Fig. 3c, the magnetic field inhibition rate varied on day 9 depending on the area of the bottom of the container. The number of clumped exposed cells in an area of 55 cm^2^ was significantly higher than that in an area of 25 cm^2^, but no significant difference was found between the number of clumped exposed cells and those in the nonexposed control group. During this period, the inhibition of suspended tumor cells disappeared, indicating that the distance between cell clusters expanded and cell growth accelerated. On the contrary, in the 25-cm^2^ culture flask, the difference in the number of cells between the exposed and control groups increased significantly, that is, the inhibition rate increased. This indicated that the tumor cells recovered contact inhibition under magnetic field exposure. Hence, the inhibition of the magnetic field required close contact between cells.
2. The experiment with adherent cells showed that the tumor cells were more susceptible to magnetic inhibition using conditioned media more suitable for cell growth. As shown in Fig. 1b and c, the number of tumor cells in the conditioned medium of the unexposed control group was significantly higher than in the fresh medium, while the number of tumor cells in the conditioned medium of the exposed group was significantly lower than in the fresh medium (the magnetic inhibition effect was significantly higher in the infusion group than in the change group). It was suggested that the conditioned medium was suitable for not only the exponential growth of cells in the absence of the magnetic field but also the communication between cells, which increased the contact inhibition effect under the magnetic field. Hence, the magnetic field inhibition rate depended on the stability of the environment to extend the contact inhibition.
3. We determined that certain substrates in the conditioned media also contributed to magnetic inhibition. The inhibition was observed when the infusion group (A549) conditioned medium was transferred to unexposed cells after magnetic field exposure, demonstrating the presence of inhibition signals in the medium. The suspended tumor cells were less sensitive to magnetic inhibition compared with the adherent tumor cells. Also, the cell aggregation was inevitably affected during the transfer of the conditioned media. Therefore, more design and consideration were required for the suspension conditional media transfer of tumor cells.

In a xenogeneic cell study in which the conditioned media of normal cells 293 T and tumor cells A549 were exchanged and transferred to unexposed cells after magnetic field exposure, the tumor cells showed inhibition while the normal cells did not show significant inhibition. It indicated that certain substrates interacting with the magnetic field were ubiquitous in cells sensitive to tumor cells. Figure 5c and d indicates that these substrates also existed in nontumor cells and did not inhibit the growth of nontumor cells. It was speculated that the contact inhibition ability of nontumor cells was not silenced, and hence the growth of nontumor cells was not inhibited by these substances. The number of no-transfer control cells was higher than that in the transfer group, possibly because the A549 condition medium was not suitable for the growth of nontumor cells. Based on the results of reduced magnetic inhibition after suspension cell dispersion or cluster separation, it was speculated that the substrates might be related to intercellular communication and cell contact inhibition. The intercellular signal increased and the contact inhibition disappeared when cells were suspended and separated or clumps were too far apart. Hence, the cells secreted one or more substrates, and the low-frequency magnetic fields interacted with cells and substrates to specifically inhibit tumor cells.

The relationship between relevant substances, conditioned media, and contact inhibition needs further experimental verification.

4. Our low-frequency magnetic field in vitro experiments had a common point that the magnetic inhibition efficiency increased with the increase in exposure time when the cells clustered and the culture environment was stable (infusion group conditioned medium), which was consistent with most current reports. Meanwhile, most reports also suggested that the magnetic field intensity positively correlated with the magnetic efficiency. Hence, the magnetic suppression efficiency positively correlated with the exposure time of magnetic field intensity^2,3,19,20^.

For the low-frequency field exposure, Trypan blue staining revealed no obvious cell staining and no significant difference in cell activity, which might be related to the fact that cell death was not observed due to direct staining after the treatment. It indicated that the magnetic field did not directly kill cells, but continuously induced apoptosis; therefore, the cells were not stained with Trypan blue. Other reports showed that the intracellular caspase-3 activity was upregulated and the membrane integrity decreased after magnetic field exposure^2,3^. Some reports revealed that some tumor cells had staining efficiency^19^, which might be related to cell type and detection time selection. Annexin V staining also showed a progressive magnetic inhibition. However, the effect varied by cell type^2^.

The specific effect of magnetic fields on signaling in the cellular environment is still unclear. The signaling pathway involved in the magnetic field may be related to contact inhibition and epigenetic inheritance. Prompted by cellular signals^1,22,27–29^, we presume the presence of electrical signals between cells and calcium ions, which are the second messenger associated with cell division. Several fluorescent probe experiments showed that the change in membrane potential was related to the exposure to the magnetic field, and the membrane potential reaction was different between adherent and suspended cells. The membrane potential of adherent cells tended to be hyperpolarized under the magnetic field. The membrane potential of clustered suspended tumor cells tended to depolarize under the magnetic field. This might be related to the cell growth and the associated intercellular communication response under the magnetic field. As shown in Fig. 3c and e, suspended and isolated Raji cells grew faster under the magnetic field. The findings of Yong Zhou's team^27^ explained this phenomenon. The cell growth rate increased during depolarization, which involved the nanoaggregation of k-Ras switches on the membrane surface. The suspended tumor cells dispersed with each other, the intercellular signaling by certain substances increased, and the magnetic field − induced cell contact inhibition disappeared. Meanwhile, the cell membrane potential of suspended tumor cells was depolarized, which accelerated cell growth, and the inhibition decreased or disappeared. The intracellular calcium signal and the membrane potential with the change in the magnetic field were relatively synchronized (except A549). We hypothesized that the calcium ion concentration was associated with the membrane potential on magnetic field exposure. In addition, only 293 T cells showed significant differences in the calcium ion concentration when exposed to the magnetic field. This was possibly related to the hypermethylation of the tumor-associated calcium signaling network^30^. At present, the relationship between calcium ion concentration and membrane potential and the inhibition signal of the magnetic field is not clear. In addition, the intracellular pH of A549 cells increased under the magnetic field. This might be related to the fact that A549 cells were more sensitive to the magnetic field. Also, this might be the reason why the calcium change in A549 cells was not synchronized with the membrane potential.

This study had certain limitations. In the experiments on suspended tumor cells, we used a pipette to destroy the cluster structure, and we could not keep the cells separated all the time during magnetic field exposure. In addition, we could not maintain cell aggregation during the transfer to larger containers every 3 days. In detecting ions and membrane potential, we knew that the membrane potential was related to magnetic field exposure. However, the kit could only measure the state of a certain period, and hence synchronous real-time detection is needed to clarify the specific relationship. Moreover, the changes in the membrane potential also indicated the changes in intracellular signals. Therefore, it was speculated that the membrane potential could be used as the means to verify the inhibitory effect of the magnetic field on tumor cells.

Combining the aforementioned four key properties of the inhibitory effect of the magnetic field on cells might serve as a good adjuvant anticancer modality. At present, magnetic field therapy has gained increasing attention. Moreover, the universality of cell signals with magnetic field exposure and the broad spectrum of tumors render it an excellent methodology for treatment and prognosis. Our findings, along with other reports, further revealed the potential of magnetic field therapy. Our next study will focus on the mechanisms involved in magnetic fields, starting with substrates.

## Methods

### Test kit

A Calbryte 520 AM calcium probe kit (item no. 20650) was purchased from AATBioquest (USA). Hanks' buffer with 20 mM HEPES (item no. 20011) and DiBAC4 (3) membrane potential fluorescent probe (item no. 21411) were also purchased from AATBioquest. A sodium ion fluorescent probe SBFI (item no. 18764) was purchased from Cayman Company (USA). A potassium ion fluorescent probe PBFI (item no. 21602) was purchased from the Cayman Company. Pluronic F-127 (ST501-10G) was purchased from Beyotime Biotechnology.

### Cell culture

293 T cells, Hepg2 cells, and A549 cells were obtained from the Cell Bank of the Chinese Academy of Sciences (Shanghai, China). The cell lines were cultured in DMEM (Biological Industries, Israel) with 10% fetal bovine serum (FBS, ExCell Bio, China) and 1% penicillin–streptomycin (P/S, Industries, Israel). The cells were incubated at 37 °C in the presence of 5% CO_2_. All operations were conducted inside the vertical-flow clean bench.

Raji cells were cultured in the RPMI1640 medium (Biological Industries) with 10% FBS and 1% P/S. The cells were incubated at 37 °C in the presence of 5% CO_2_. All operations were conducted inside the vertical-flow clean bench.

### Magnetic field exposure and characteristics

**Figure 6:**
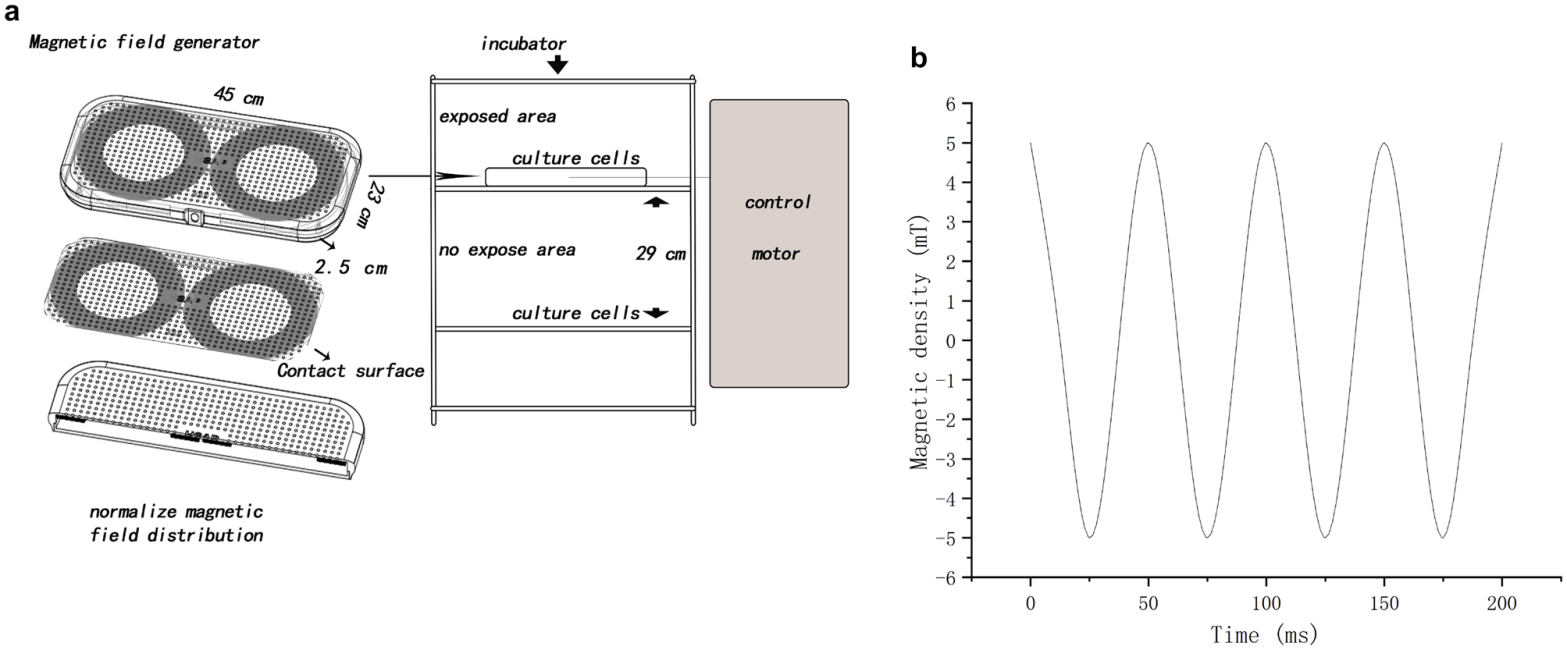
Magnetic field exposure system. (**a**) Schematic diagram of the magnetic field exposure system. Details are described in the Methods section. (**b**) Magnetic field waveform.

The self-made magnetic field generator converted electrical signals into magnetic field signals through an enameled copper wire (Fig. 6, Fig. S4). The shell was made of acrylonitrile butadiene styrene plastic material with specifications: 450 × 230 × 25 (L × D × H, mm^3^). The magnetic field output could be changed by adjusting the frequency and amplitude of the power supply voltage of the equipment to the magnetic field–generating device. The magnetic field–generating device was placed in an incubator, and the magnetic field was measured with a Gauss meter (TES 1393; TES Electrical Electronic Corp, Taiwan). The cells in the nonexposed group were placed in the same incubator (Thermo Scientific, USA). The direction of the magnetic field was perpendicular to the magnetic field generator. During the whole experiment, the intensity of the stray magnetic field in each incubator was less than 0.02 mT (0–0.02 mT), and the temperature was adjusted to 37 ± 0.18 °C.

### Cell counting

Adherent cells: The supernatant was collected in a centrifuge tube and washed twice with normal saline. The cleaned supernatant was extracted, added to the centrifuge tube, and then digested with trypsin (optimal digestion time varied from cell to cell: 293 T, 1 min; Hepg2, 1 min; A549, 2 min). The digestion was terminated in the medium, and all cells were added to the centrifuge tube. Then, the hole was cleaned with normal saline many times and added to the centrifuge tube. At this time, the centrifuge tube contained all cells. Whether cells were left in the hole was observed under the microscope.

Suspended cells: All cell suspensions were placed in a centrifuge tube. The holes were washed with normal saline several times and placed in the centrifuge tube. At this time, the centrifuge tube contained all cells. The pores were observed for cell residue under the microscope.

Counting: The cells were centrifuged, resuspended in an appropriate medium, diluted partially, and stained with Trypan blue. Then, 10 µL of it was injected through the cover glass.

Calculation formula: Number of cells/4 × volume × dilution ratio × 10^4^.

The cells in each group were counted three times for a total of three groups.

### pH value detection 

The membrane-permeable fluorescence dye BCECF AM and pH-sensitive fluorescent probe 2’,7’-bis-(2-carboxyethyl)-5-carboxyfluorescein (Beyotime Biotechnology, China) were used to assess the intracellular pH value. The cells were cultured in 96-well plates and exposed to a 5-mT, 20-Hz magnetic field for 2 h on the first 2 days. On day 3, the cells were resuspended in 100 µL of 25 µM BCECF AM in 0.04% Pluronic F-127 working solution and incubated in a CO_2_ incubator at 37 ± 0.18 °C for 1 h. The supernatant was replaced with HHBS buffer (AAT Bioquest, USA), and the cells were subjected to magnetic field exposure for 2 h. The supernatant was then replaced with PBS, and the pH values were determined using FITC flow cytometry (FACS Calibur, BD).

### Intracellular calcium concentration assays

The membrane-permeable fluorescence dye Calbryte 520 AM (AAT Bioquest) was used to assess the intracellular calcium concentration. The cells were cultured in 96-well plates and exposed to a 5-mT, 20-Hz magnetic field for 2 h on the first 2 days. On day 3, the cells were resuspended in 100 µL of 5 µM Calbryte 520 AM in 0.04% Pluronic F-127 working solution and incubated in a CO_2_ incubator at 37 ± 0.18 °C for 1 h. The supernatant was replaced with HHBS buffer (AAT Bioquest), and the cells were exposed to the magnetic field for 2 h. The supernatant was then replaced with PBS, and the intracellular calcium concentration values were determined using FITC flow cytometry (FACS Calibur).

### Determination of membrane potential of cells

The membrane potential was determined using the potential-sensitive fluorescence dye bis-(1,3-dibutylbarbituric acid) trimethine oxonol (DiBAC4) (3) (AAT Bioquest). The fluorescent dye DiBAC4 (3) permeated into the depolarized cells with high membrane potential, leading to an increase in the intracellular fluorescence intensity. DiBAC4 (3) was discharged from the hyperpolarized cells, and the intracellular fluorescence intensity decreased. The cells were cultured in 96-well plates and exposed to a 5-mT, 20-Hz magnetic field for 2 h on the first 2 days. The cells were cultured in 100 µL of HHBS. On day 3, the cells were resuspended in 100 µL of 10 µM DIBAC4 (3) AM in 0.04% Pluronic F-127 working solution and incubated in a CO_2_ incubator at 37 ± 0.18 °C for 1 h. The supernatant was replaced with HHBS buffer (AAT Bioquest), and the cells were exposed to the magnetic field for 2 h. The supernatant was then replaced with PBS, and the cell membrane potentials were determined using FITC flow cytometry (FACS Calibur).

### Sodium ion detection

The cells were cultured in 96-well plates and exposed to a 5-mT, 20-Hz magnetic field for 2 h on the first 2 days. On day 3, the medium was replaced with 100 µL of HHBS and 100 µL of 10 µM SBFI AM (Cayman Chemical) in a 0.04% Pluronic F-127 working solution. The supernatant was replaced with HHBS buffer (AAT Bioquest) after 4-h incubation in a CO_2_ incubator at 37 ± 0.18 °C, and the cells were exposed to the magnetic field for 2 h. The sodium ion was determined at 330/80 excitation and 528/20 emission using a microplate tester.

### Potassium ion detection

The cells were cultured in 96-well plates and exposed to a 5-mT, 20-Hz magnetic field for 2 h on the first 2 days. On day 3, the medium was replaced with 100 µL of HHBS and 100 µL of 10 µM PBFI AM (Cayman Chemical) in a 0.04% Pluronic F-127 working solution. After 4-h incubation in a CO_2_ incubator at 37 ± 0.18 °C, the supernatant was replaced with HHBS buffer (AAT Bioquest), and the cells were exposed to the magnetic field for 2 h. The potassium ion determination was conducted at 330/80 excitation and 528/20 emission using a microplate tester.

### Trypan blue dyeing

293 T, Hepg2, and A549 cells in the logarithmic growth phase were trypsin digested and harvested. Raji cells were collected directly. Following centrifugation, the cells were stained with the vital dye Trypan blue. The live cells were counted under an inverted light microscope (Carl Zeiss, Germany). The cell growth inhibition rates were determined, and representative curves were plotted. Inhibition rate (%) = (number of cells in the control group − number of cells in the magnetic field group) / number of cells in the control group × 100%.

### Statistical analysis

All data were expressed as mean ± standard error of the mean, and one-way analysis of variance was used for comparison between multiple groups. The Student *t* test was used to compare the treated versus untreated groups. A *P* value < 0.05 indicated a statistically significant difference. The confirmatory results at *P* < 0.05 were obtained from repeating studies at least two times. Each set of experiments had three parallel samples and each experiment was repeated three times.

## Supplementary Information

Supplementary Information.

## Data Availability

All data are presented in this published article and supplementary file.

## References

[1] Zhang X (2010). Magnetic fields at extremely low-frequency (50 Hz, 0.8 mT) can induce the uptake of intracellular calcium levels in osteoblasts. Biochem. Biophys. Res. Commun..

[2] Li J (2014). Natural static magnetic field-induced apoptosis in liver cancer cell. Electromagn Biol. Med..

[3] Koh EK (2008). A 60-Hz sinusoidal magnetic field induces apoptosis of prostate cancer cells through reactive oxygen species. Int. J. Radiat. Biol..

[4] Cameron IL (2005). Therapeutic electromagnetic field (TEMF) and gamma irradiation on human breast cancer xenograft growth, angiogenesis and metastasis. Cancer Cell Int..

[5] de Seze R (2000). Effects of 100 mT time varying magnetic fields on the growth of tumors in mice. Bioelectromagnetics.

[6] Novikov VV, Novikov GV, Fesenko EE (2009). Effect of weak combined static and extremely low-frequency alternating magnetic fields on tumor growth in mice inoculated with the Ehrlich ascites carcinoma. Bioelectromagnetics.

[7] Tatarov I (2011). Effect of magnetic fields on tumor growth and viability. Comp. Med..

[8] Tofani S (2002). Increased mouse survival, tumor growth inhibition and decreased immunoreactive p53 after exposure to magnetic fields. Bioelectromagnetics.

[9] Tofani S (2001). Static and ELF magnetic fields induce tumor growth inhibition and apoptosis. Bioelectromagnetics.

[10] Williams CD (2001). Therapeutic electromagnetic field effects on angiogenesis and tumor growth. Anticancer Res..

[11] Yamaguchi S (2006). Effects of pulsed magnetic stimulation on tumor development and immune functions in mice. Bioelectromagnetics.

[12] Berg H (2010). Bioelectromagnetic field effects on cancer cells and mice tumors. Electromagn Biol. Med..

[13] Liang Y (1997). Enhanced potency of daunorubicin against multidrug resistant subline KB-ChR-8-5-11 by a pulsed magnetic field. Anticancer Res..

[14] Omote Y (1990). Treatment of experimental tumors with a combination of a pulsing magnetic field and an antitumor drug. Jpn. J. Cancer Res..

[15] Xu L (2015). Synergistic inhibitory effect of static magnetic field and antitumor drugs on Hepa1-6 cells. Sheng Wu Gong Cheng Xue Bao.

[16] Gellrich D, Becker S, Strieth S (2014). Static magnetic fields increase tumor microvessel leakiness and improve antitumoral efficacy in combination with paclitaxel. Cancer Lett..

[17] El-Bialy NS, Rageh MM (2013). Extremely low-frequency magnetic field enhances the therapeutic efficacy of low-dose cisplatin in the treatment of Ehrlich carcinoma. Biomed. Res. Int..

[18] Gellrich D (2018). Modulation of exposure to static magnetic field affects targeted therapy of solid tumors In Vivo. Anticancer Res..

[19] Crocetti S (2013). Low intensity and frequency pulsed electromagnetic fields selectively impair breast cancer cell viability. PLoS ONE.

[20] Raylman RR, Clavo AC, Wahl RL (1996). Exposure to strong static magnetic field slows the growth of human cancer cells in vitro. Bioelectromagnetics.

[21] Wang T (2011). Involvement of midkine expression in the inhibitory effects of low-frequency magnetic fields on cancer cells. Bioelectromagnetics.

[22] Buckner CA (2015). Inhibition of cancer cell growth by exposure to a specific time-varying electromagnetic field involves T-type calcium channels. PLoS ONE.

[23] Wang MH (2021). Effect of extremely low frequency electromagnetic field parameters on the proliferation of human breast cancer. Electromagn. Biol. Med..

[24] Kim SH (2006). Toxicity bioassay in Sprague–Dawley rats exposed to 20 kHz triangular magnetic field for 90 days. Bioelectromagnetics.

[25] Ashdown CP (2020). Pulsed low-frequency magnetic fields induce tumor membrane disruption and altered cell viability. Biophys. J..

[26] Watson JM, Parrish EA, Rinehart CA (1998). Selective potentiation of gynecologic cancer cell growth in vitro by electromagnetic fields. Gynecol. Oncol..

[27] Zhou Y (2015). Signal transduction. Membrane potential modulates plasma membrane phospholipid dynamics and K-Ras signaling. Science.

[28] Berzingi S, Newman M, Yu HG (2016). Altering bioelectricity on inhibition of human breast cancer cells. Cancer Cell Int..

[29] Leslie TK (2019). Sodium homeostasis in the tumour microenvironment. Biochim. Biophys. Acta Rev. Cancer.

[30] Wang XX (2017). Large-scale DNA methylation expression analysis across 12 solid cancers reveals hypermethylation in the calcium-signaling pathway. Oncotarget.

