# Supplementary material for: Effects of extremely low frequency electromagnetic fields on the tumor cell inhibition and the possible mechanism
Source: Sci Rep. 2023 Apr 28;13:6989. doi: 10.1038/s41598-023-34144-5 (PMC10147919; doi:10.1038/s41598-023-34144-5)
Supplement: Supplementary file 1 — Supplementary Information. [file 41598_2023_34144_MOESM1_ESM.pdf]

Effects of extremely low frequency electromagnetic fields on the tumor cell inhibition and the possible mechanism

Jie Sun[1,2,3], Yingying Tong[1,2,3], Yu Jia[1,2,3], Xu Jia[1,2,3], Hua Wang[4], Yang Chen[1,2,3], Jiamin Wu[5], Weiyang Jin[5], Zheng Ma[6], Kai Cao[6], Xiangdong Li[6], Zhonglin Chen[6], Guanghua Yang[1,2,3,*]

https://orcid.org/0000-0002-5749-4887

[1] International Research Center for Biological Sciences, Ministry of Science and Technology, Shanghai Ocean University, Shanghai 201306, China.

[2] National Aquatic Animal Pathogen Collection Center, Shanghai Ocean University, Shanghai 201306, China.

[3] Aquatic Animal Genetics and Breeding Center, Shanghai Ocean University, Shanghai 201306, China.

[4] Shanghai telebio biomedical co., ltd,Shanghai, China.

[5] Zhejiang Huayi Health Industry Development Co., Ltd,Zhejiang,China.

[6] Huisi Anpu Medical System Co., Ltd,Qinhuangdao,China

[*] e-mail:ghyang119@163.com

Supporting information

Fig S1. Flow cytometry (pH).

expose:

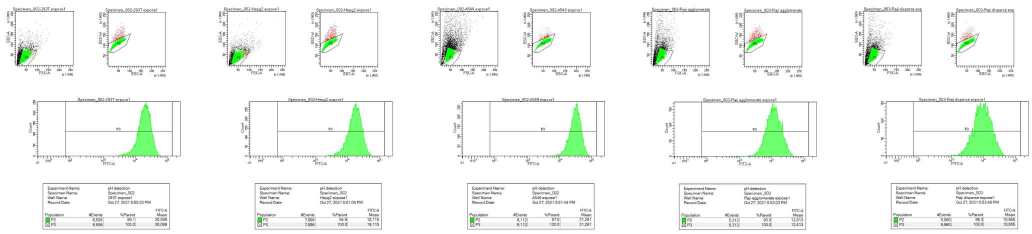

no expose:

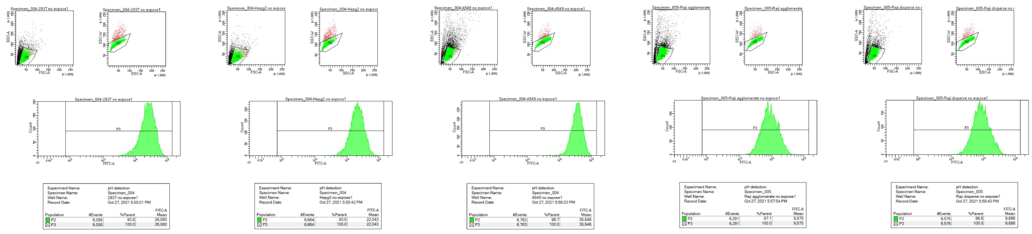

The original data of flow cytometry for pH detection were derived from BD flow cytometry. As supplementary data of Fig 4c, this figure mainly provides detailed information such as gating channel data during detection.

Fig S2. Flow cytometry (calcium ions).

expose:

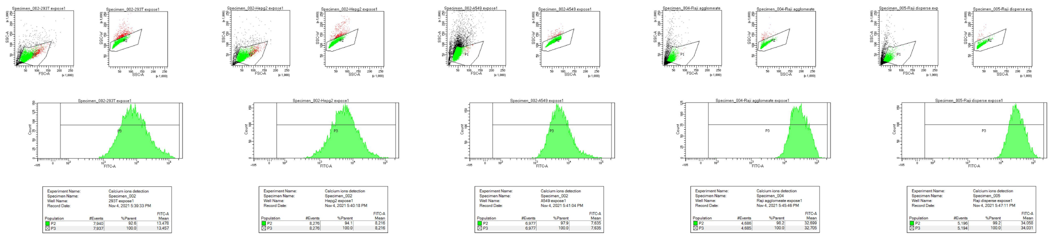

no expose:

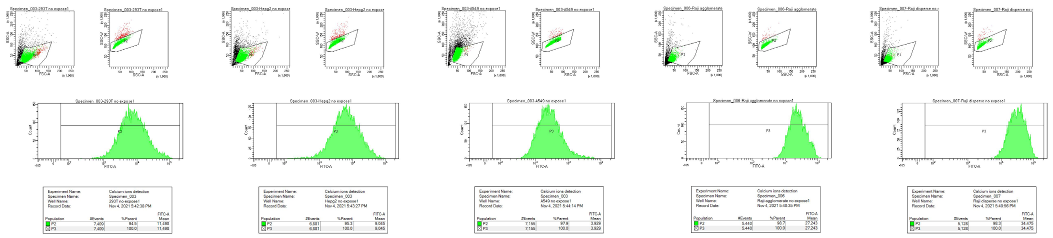

The original data of flow cytometry for calcium ion detection were derived from BD flow cytometry. As supplementary data of Fig 4d, this figure mainly provides detailed information such as gating channel data during detection.

Fig S3. Flow cytometry (membrane potential).

expose:

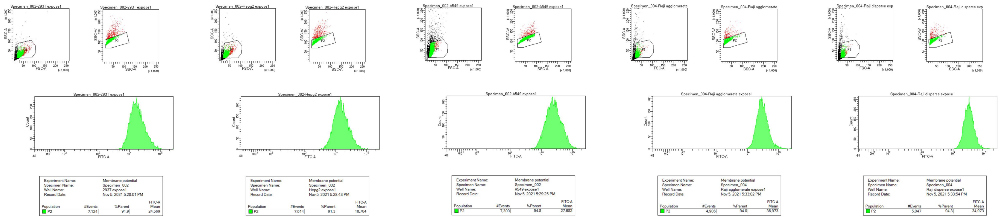

no expose:

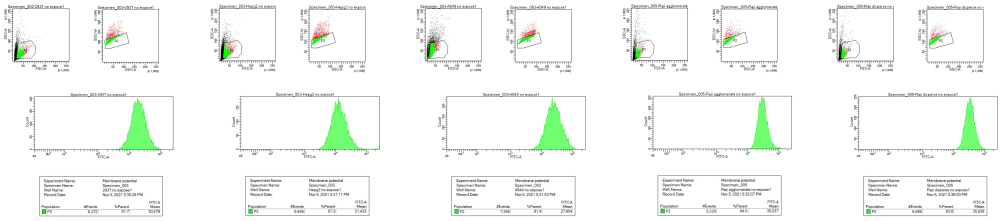

The original data of flow cytometry for membrane potential detection were derived from BD flow cytometry. As supplementary data of Fig 4e, this figure mainly provides detailed information such as gating channel data during detection.

Fig S4. The design drawing of magnetic field generator.

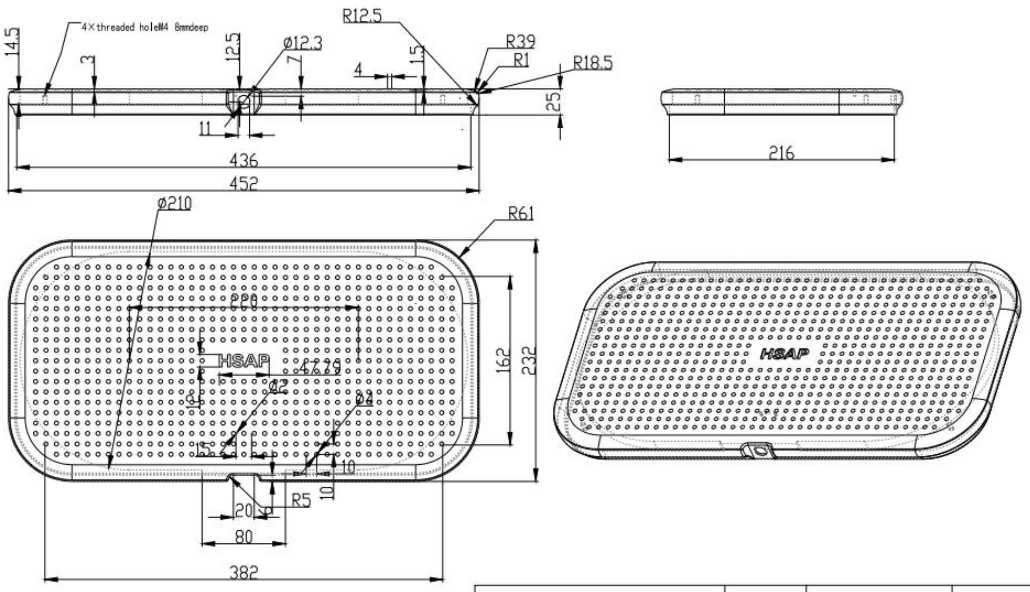

Requirements:
1. Polish the surface and remove burrs
2, spray paint matte white
3. This is page 1, with 3 pages in total

PMR Treatment box -V3.0			material	ABS plastic (CNC machining)	UNIT mm
			proportion		
		2021.03.17	Huisi Anpu Medical System Co., Ltd		

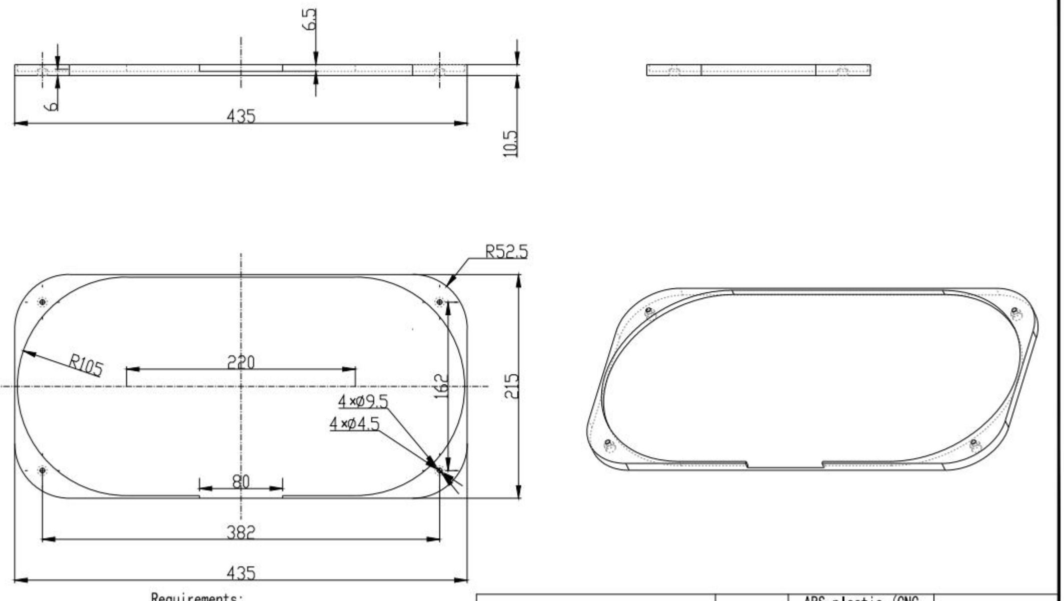

Requirements:
1. Polish the surface and remove burrs
2, spray paint matte white
3. This is page 2, with 3 pages in total

PMR Treatment box -V3.0			material	ABS plastic (CNC machining)	UNIT mm
			proportion		
		2021.03.17	Huisi Anpu Medical System Co., Ltd		

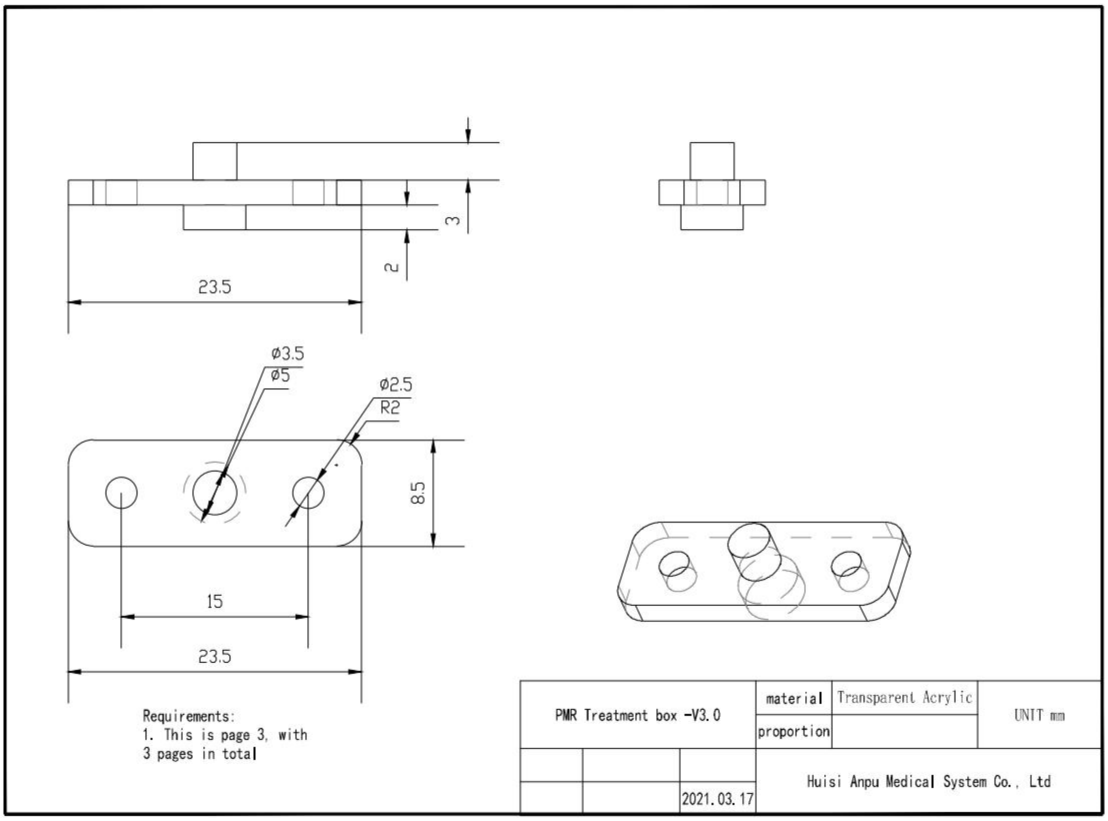

The magnetic field generator is customized and developed by Huisi Anpu Medical System Co., Ltd,Qinhuangdao,China.This is the design drawing of our magnetic field generator.

Fig S5. Double-blind experimental replication of experiment 1, as requested by the reviewer.

Day / Number (10^5)	No expose infusion			Expose infusion			No expose change			Expose change		
	Rpt 1	Rpt 2	Rpt 3	Rpt 1	Rpt 2	Rpt 3	Rpt 1	Rpt 2	Rpt 3	Rpt 1	Rpt 2	Rpt 3
1 day	2.50	2.45	2.62	2.85	2.48	2.50	2.08	2.50	2.25	2.05	2.23	2.15
2 day	8.50	7.80	8.35	8.20	7.90	8.05	7.05	7.40	7.80	8.45	7.80	8.15
3 day	18.90	18.80	18.50	18.40	18.40	18.80	19.20	19.40	20.00	19.60	18.20	19.30

Table 1, 293T cells blinded test data

Day / Number (10^5)	No expose infusion			Expose infusion			No expose change			Expose change		
	Rpt 1	Rpt 2	Rpt 3	Rpt 1	Rpt 2	Rpt 3	Rpt 1	Rpt 2	Rpt 3	Rpt 1	Rpt 2	Rpt 3
1 day	1.50	1.58	1.60	1.43	1.28	1.33	1.33	1.63	1.55	1.63	1.23	1.43
2 day	4.70	5.00	4.90	3.65	3.90	3.75	4.50	4.45	4.65	4.00	3.60	3.85
3 day	11.50	10.70	11.30	8.20	8.10	7.80	9.40	9.00	9.80	7.20	8.10	8.20

Table 2, A549 cells blinded test data

Day / Number (10^5)	No expose infusion			Expose infusion			No expose change			Expose change		
	Rpt 1	Rpt 2	Rpt 3	Rpt 1	Rpt 2	Rpt 3	Rpt 1	Rpt 2	Rpt 3	Rpt 1	Rpt 2	Rpt 3
1 day	3.08	3.00	3.18	2.43	2.80	2.58	2.70	2.73	2.70	2.50	2.35	2.38
2 day	8.90	9.35	9.35	6.75	7.25	7.10	8.15	7.90	8.30	6.60	6.55	7.00
3 day	21.40	20.70	21.60	18.70	19.70	18.50	20.50	21.10	20.30	18.00	19.40	19.50

Table 3, Hepg2 cells blinded test data

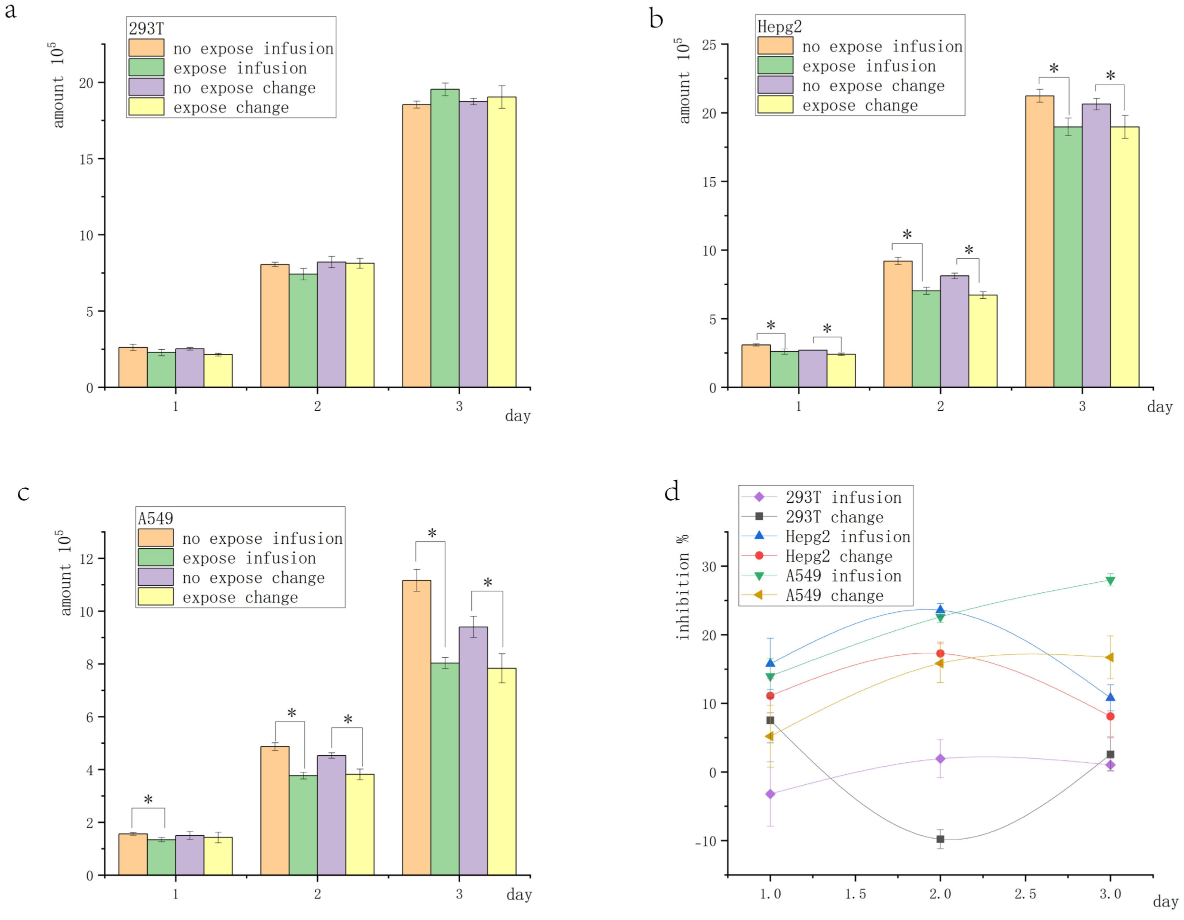

Difference in the environment (conditioned medium) before exposure affected the inhibitory effect of the magnetic field on adherent cells. (a) No significant difference was found in the number of 293T cells in 3 days. (b) Number of Hepg2 cells in the unexposed and exposed groups was significantly different. (c) Number of A549 cells in the unexposed and exposed groups was significantly different. (d) Cell inhibition curve. The cell inhibition rate in the infusion group was more obvious than that in the change group, and the normal cell inhibition rate was not of statistical significance (*$P < 0.05$, versus the no-exposure control group).

The results are consistent with the manuscript.
